# The patient's perspective on radiation for rectal cancer: Initial expectations versus actual experience

**DOI:** 10.1002/cam4.6541

**Published:** 2023-09-29

**Authors:** Basil H. Chaballout, Eric M. Chang, Narek Shaverdian, Percy P. Lee, Phillip J. Beron, Michael L. Steinberg, Ann C. Raldow

**Affiliations:** ^1^ University of South Carolina School of Medicine Greenville Greenville South Carolina USA; ^2^ Oregon Health and Science University School of Medicine Portland Oregon USA; ^3^ Memorial Sloan Kettering Cancer Center New York New York USA; ^4^ UCLA Department of Radiation Oncology Los Angeles California USA; ^5^ Cancer Treatment Centers of America Phoenix Arizona USA

**Keywords:** cancer education, cancer prevention, cancer risk factors, radiotherapy

## Abstract

**Background:**

The aim of this study was to compare patient perceptions of radiotherapy (RT) before and after treatment to better inform future patients and providers.

**Methods:**

Seventy‐eight consecutive patients with rectal adenocarcinoma treated with neo‐ or adjuvant chemoradiation, surgical resection, and adjuvant chemotherapy from 2009 to 2018 and who were without recurrence were included. Patients were surveyed ≥6 months after ileostomy reversal or ≥3 months after adjuvant chemotherapy. The survey assessed patients' baseline knowledge and fears of RT, how their short‐ and long‐term side effects compared with initial expectations, and how their experiences compared for each modality (RT, surgery, and chemotherapy).

**Results:**

Forty patient‐responses were received. Before treatment, 70% of patients indicated little to no knowledge of RT, though 43% reported hearing frightening stories about RT. The most commonly top‐ranked fears included organ damage (26%), skin burns (14%), and inability to carry out normal daily activities (10%). Eighty percent reported short‐term effects of RT to be less than or as expected, with urinary changes (93%), abdominal discomfort (90%), and anxiety (88%) most commonly rated as less than or as expected. 85% reported long‐term effects to be less than or as expected, with pain (95%), changes to the appearance of the treated area (85%), and dissatisfaction with body image (80%) most commonly rated as less than or as expected. Surgery was most commonly rated as the most difficult treatment (50%) and most responsible for long‐term effects (55%). RT was least commonly rated as the most difficult treatment (13%), and chemotherapy was least commonly rated as most responsible for long‐term effects (13%).

**Conclusions:**

The majority of patients indicated short‐ and long‐term side effects of RT for rectal cancer to be better than initial expectations. In the context of trimodality therapy, patients reported RT to be the least difficult of the treatments.

## 1 INTRODUCTION

The treatment of locally advanced rectal cancer involves trimodality therapy, historically consisting of neoadjuvant chemoradiation, total mesorectal excision (TME) surgery, and adjuvant chemotherapy.^1^ Particularly relevant to the patient experience, each of these modalities can be associated with significant short‐term and long‐term effects, which may cause varying levels of distress based on patients' perceptions and values. Qualitative studies have evaluated sources of distress and expectations of functional outcomes in patients undergoing surgery for rectal cancer, though how these perceptions are considered relative to the experience of the other arms of treatment, neoadjuvant chemoradiation, and adjuvant systemic therapy, remains unclear.^2^, ^3^ Furthermore, as the modalities may cause some similar long‐term effects, little is known how patients attribute their long‐term outcomes to each modality at the conclusion of treatment. Better understanding of the patients' perceptions of each arm of treatment would help inform future patients as well as aid physicians in helping patients manage expectations for the duration of treatment.

Neoadjuvant chemoradiation therapy is a standard of care for patients with locally advanced rectal cancer, with aim of reducing risk of local recurrence and increasing rates of sphincter‐preserving surgery in patients with low‐lying tumors.^1^ Patients undergoing chemoradiation may still experience significant treatment‐related acute and late effects potentially impacting both their functional outcome and quality of life.^4^ We evaluated the baseline fears of patients undergoing neoadjuvant chemoradiation for rectal cancer prior to treatment and how patients' experiences of the short‐ and long‐term effects of neoadjuvant chemoradiation for rectal cancer compare with prior expectations at the completion of treatment. In addition, we retrospectively evaluated relative perceptions of the different arms of trimodality treatment (neoadjuvant chemoradiation, surgery, adjuvant chemotherapy) in patients undergoing treatment for locally advanced rectal cancer.

## 2 METHODS

### 2.1 Patients and treatments

Consecutive patients with adenocarcinoma of the rectum treated with neo‐ or adjuvant chemoradiation, surgical resection, and adjuvant chemotherapy at the University of California Los Angeles Department of Radiation Oncology from 2009 to 2018 and who were without recurrence were included. Patients were surveyed ≥6 months after ileostomy reversal or ≥3 months after adjuvant chemotherapy, whichever was later. Patients with any prior history of radiation were excluded. Baseline characteristics of the patients included in the study are shown in Table 1.

**TABLE 1:** Baseline patient characteristics.

Demographic	Number of patients (*N* = 40)	Percentage
Age (years)		
Median age	68	
Range	39–89	
Sex		
Male	19	47.5%
Female	21	52.5%
Highest education level completed		
Highschool	9	22.5%
College	15	37.5%
Postgraduate	15	37.5%
Stage		
I	2	5%
II	8	20%
III	30	75%
Radiotherapy		
Median dose	50.4 Gy	
Dose range	45–54 Gy	
3DCRT	21	52.5%
IMRT	19	47.5%
Neoadjuvant	35	87.5%
Concurrent chemo		
Xeloda	34	85%
5‐FU	5	12.5%
Other	1	2.5%
Surgery		
LAR	26	65%
APR	10	25%
Other	4	10%
pCR	14	40% (of those receiving neoadjuvant chemoRT)
Chemotherapy		
FOLFOX	7	17.5%
Xelox	17	42.5%
Cape	12	30%
None	4	10%
Median cycles	6	
Range of cycles	2–11	

### 2.2 Instruments and measures

The survey was designed to assess patients' baseline knowledge and fears of chemoradiation and asked them to compare their experiences of the short‐ and long‐term effects with initial expectations (see Data S1). The survey additionally assessed how patients perceive RT relative to the other arms of their treatment (surgery and chemotherapy). Measures of short‐ and long‐term effects of treatment in the survey were adapted from validated patient‐reported questionnaires, including the European Organization for Research and Treatment of Cancer QLQ‐ANL27, CR29, and C30 questionnaires.

After approval by the institutional review board at the study institution, the survey was administered to 78 patients over a 6 month period. Surveys were either mailed or provided to patients follow‐up. Most of the questions were asked with the following answer choice types depending on question:

The scale for questions asking for post‐treatment patient experience compared with pre‐treatment patient perception was as follows:

1. what I had expected.
2. slightly less than I had expected.
3. significantly less than I had expected.
4. slightly more than I had expected.
5. significantly more than I had expected.

The scale for true or false questions regarding patient experiences was as follows:

1. Definitely false.
2. Mostly false.
3. Neither true nor false.
4. Mostly true.
5. Definitely true.
6. Not applicable/I did not hear stories from friends and family.

The remainder of the questions were regarding patient demographics or patient preferences and required specific answer choices. For example, the question that asked where patients' main sources of pre‐treatment RT knowledge came from had the following answer choices:

1. Internet articles or blogs.
2. Scientific articles
3. Friends/family
4. Physicians
5. Other (please specify): __________________________

Answer choices were plotted and basic statistics were analyzed.

## 3 RESULTS

### 3.1 Patient clinical characteristics

The response rate was 51% (40/78). The median age was 68 years (range 39–89). More than half of responders were female (53%) and had completed college (37.5%) or post graduate education (37.5%). The clinical stage distribution was 5% stage I (upstaged at surgery), 20% stage II, and 75% stage III. The majority underwent neoadjuvant (88%) chemoradiation with capecitabine (85%); 53% were treated with 3D conformal RT (3DCRT) and 47% intensity‐modulated RT (IMRT, median total dose 50.4 Gy, range 45–54 Gy). Surgical resections were as follows: 65% low anterior resection, 25% abdominal perineal resection, and 10% other. The most common adjuvant chemotherapy was capecitabine/oxaliplatin (43%) for median duration of 6 cycles. Median follow‐up at survey was 41 months (range 4–107 months), and was calculated using a standard median function. See Table 1 for the remaining baseline characteristics.

### 3.2 Baseline perceptions and knowledge of RT

Only 15% of respondents (6 patients) reported having a lot of prior knowledge of RT at the time of diagnosis, while 70% of respondents (28 patients) reported that they had little to no baseline knowledge. The most common source of information for those patients with some baseline knowledge was friends and family (13 patients). About 43% of respondents (17 patients) reported having previously read or heard scary stories about patients having serious side effects from RT. Among those who ranked their top fears about RT, the most commonly ranked fears were: organ damage (25.83%), skin burns (14.17%), not being able to carry out normal daily activities (10%), weakness (7.5%), damage to the immune system (6.67%), pain (4.17%), diarrhea (4.17%), tiredness (2.5%), changes in appearance (2.5%), changes in ability to engage in sexual activity (1.67%), and not being able to engage in social and leisure time activities (0.83%).

### 3.3 Beliefs regarding RT post actual treatment experience

No patients found the negative stories they previous read online about RT to be definitely true or definitely false. However, 25% of patients (10 patients) found the stories to be mostly false, and 10% of patients (4 patients) found the stories to be mostly true. No patients found the stories regarding RT from friends and family to definitely be true, 2.5% of patients (1 patient) found the stories to be definitively false, 22.5% of patients found them to be mostly false, and 12.5% of patients (5 patients) found them to be mostly true. 83% of patients (33 patients) reported that the overall RT experience was less scary than predicted. Only 10% of patients (four patients) found the overall RT experience to be harder than expected. Only 10% of patients (four patients) were surprised by how severe the actual side effects from RT were. Approximately 88% of patients (35 patients) did not feel they would have been better off without radiation treatment. Additionally, 83% of patients (33 patients) agreed with this statement: “If future patients knew the read truth about radiation therapy, they would be less scared about treatment.”

### 3.4 Perceptions of acute and long‐term RT toxicity

**FIGURE 1:**
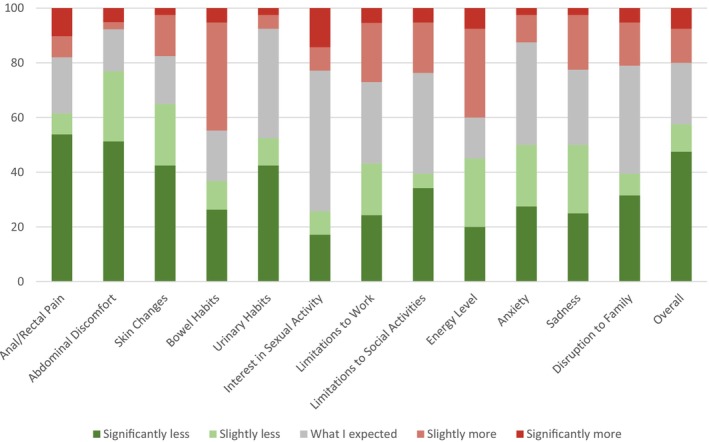
Patient‐reported assessments of how actual short‐term toxicities compared with initial expectations.

Regarding overall short‐term radiation side effects during RT, approximately 57.5% of respondents (23 patients) found the severity to be either slightly or significantly less than expected. Only 12.5% of respondents (five patients) reported slightly more than expected overall severity of short‐term RT side effects. About 8% of respondents (three patients) reported significantly more than expected overall severity of short‐term radiation side effects. The following are patient perceptions of acute toxicity broken down by system (see Figure 1):

#### 3.4.1 Gastrointestinal (GI) system

Regarding severity of anal or rectal pain experienced during RT, 7.5% of respondents (3 patients) and 52.5% of respondents (21 patients) reported the impact to be slightly or significantly less than expected respectively. Regarding short‐term RT induced bowel habit changes, 35% of respondents (14 patients) reported slightly or significantly less than expected changes, and 42.5% (17 patients) reported slightly or significantly more than expected changes. Regarding abdominal discomfort, 25% of respondents (10 patients) and 50% of respondents (20 patients) found the experience to be less than or as expected during their RT.

#### 3.4.2 Genito‐urinary (GU) system

Regarding urinary habits, 10% of respondents (4 patients) and 42.5% of respondents (17 patients) reported the changes as slightly or significantly less than expected respectively. Regarding interest in sexual activity, 45% of respondents (18 patients) reported the changes to be as expected, and 22.5% of respondents (9 patients) reported the changes as slightly or significantly less than expected.

#### 3.4.3 Overall/other systems

Regarding severity of short‐term radiation induced skin changes 22.5% of respondents (9 patients) and 42.5% of respondents (17 patients) reported slightly and significantly less than expected respectively. 35% of respondents (14 patients) found the limitations to social activities during treatment to be as expected, and 37.5% of respondents (15 patients) found them to be slightly or significantly less. Regarding energy level during treatment, 45% of respondents (18 patients) reported slightly or significantly less than expected, and 40% of respondents (16 patients) reported slightly or significantly more than expected. Regarding limitations to work activities, 27.5% of respondents (11 patients) found the changes to be as expected, and 40% of respondents (16 patients) found the changes to be slightly or significantly less than expected. Regarding disruption to friends and family, 37.5% of respondents (15 patients) reported the changes to be as expected, and 37.5% of respondents (15 patients) reported the changes as slightly or significantly less than expected. Regarding the amount of anxiety felt during RT, 37.5% of respondents (15 patients) reported the changes to be as expected, and 50% of respondents (20 patients) reported the changes as slightly or significantly less than expected. Lastly, regarding amount of sadness during treatment, 25% of respondents (10 patients) reported the changes to be as expected, and 50% of respondents (20 patients) reported the changes as slightly or significantly less than expected.

**FIGURE 2:**
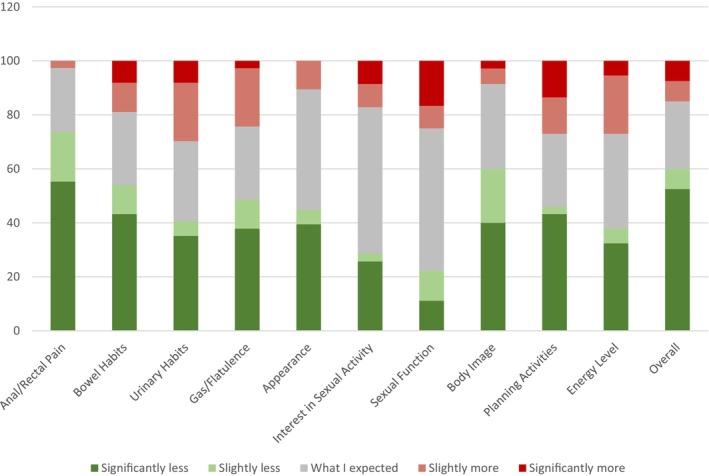
Patient‐reported assessments of how actual long‐term toxicities compared with initial expectations.

Regarding overall severity of long‐term radiation side effects during RT, 60% of respondents (24 patients) reported it as either slightly or significantly less than expected, and 15% of respondents (6 patients) reported it as either slightly or significantly more than expected. The following are patient perceptions of long‐term toxicity broken down by system (see Figure 2):

#### 3.4.4 Gastrointestinal (GI) system

Regarding severity of anal or rectal pain, 17% of respondents (7 patients) and 55% of respondents (22 patients) reported it as slightly and significantly less than expected respectively. Regarding changes to the bowel habits, 40% of respondents (16 patients) reported them to be significantly less than expected, and 25% of respondents (10 patients) reported them to be as expected. Regarding problems with flatulence, 45% of respondents (18 patients) reported them to be slightly or significantly less than expected, and 25% of respondents (10 patients) reported them to be as expected.

#### 3.4.5 Genito‐urinary (GU) system

Regarding the changes to urinary habits, 27.5% of respondents (11 patients) reported them to be as expected, and 5% of respondents (2 patients) and 32.5% of respondents (13 patients) reported them to be slightly and significantly less than expected respectively. As for interest in sexual activity, 47.5% of respondents (19 patients) reported the changes to be as expected, and 22.5% of respondents (9 patients) reported them to be significantly less than expected. As for changes to sexual function, 47.5% of respondents (19 patients) reported the changes to be as expected, and 20% of respondents (8 patients) reported them to be slightly or significantly less than expected.

#### 3.4.6 Overall/other systems

Regarding problems planning activities in advance, 40% of respondents (16 patients) reported the changes to be significantly less than expected, and 25% of respondents (10 patients) reported the changes to be as expected. As for change in appearance of area treated with radiation, 37.5% of respondents (15 patients) reported the changes to be significantly less than expected, and 42.5% of respondents (17 patients) reported them to be as expected. Regarding dissatisfaction with body image, 52.5% of respondents (21 patients) reported the effect to be slightly or significantly less than expected, and 27.5% of respondents (11 patients) reported it to be as expected.

### 3.5 Modalities

Half of the respondents (20 patients) rated surgery as the most difficult treatment modality. Of the three options of surgery, chemotherapy, and RT, only 12% of respondents (5 patients) rated RT as most difficult. Most respondents (55%, 22 patients) in fact labeled RT as the least difficult modality. When questioned which modality patients felt was most responsible for their long‐term side effects, most patients (55%, 22 patients) chose surgery. When questioned which modality was least responsible, the most selected option was RT (45%, 18 patients).

## 4 DISCUSSION

As radiotherapy continues to evolve and long‐term effects of modern therapy are continually changing, it is important to acknowledge that patient perceptions of radiotherapy may not fully be in sync with the most up‐to‐date practices. These practices include concepts such as total neoadjuvant chemotherapy (TNT) and organ preservation, and ultimately are fine‐tuning and refining treatment to maximize outcomes while minimizing long‐term side effects.^5^, ^6^, ^7^, ^8^, ^9^ Recent studies have attempted to gather a nuanced understanding of these patient perspectives and the potential discrepancy between past and modern treatment modalities. For instance, a specific focus on patient perspective of breast radiotherapy recently found that while breast RT is associated with significant fear and misconceptions, patients indicated their actual treatment experience was especially superior.^10^ Other studies have focused specifically on the long‐term sequelae of current rectal cancer RT, but have not compared these results with patient expectations.^11^ It was concluded that further studies are necessary to have a comprehensive understanding as to which treatment approach is better for each individual patient and their specific beliefs and preferences. We aimed to gather a wholistic understanding of the patient's rectal cancer treatment beliefs and experiences.

The responses acquired in the current study demonstrate that most patients (70%) came into treatment with little to no baseline knowledge of RT. Yet, nearly half (43%) previously read or heard scary stories of serious side effects. Despite this, none of the patients found these stories to be definitely true, and nearly all patients (88%) agreed that they were better off having gone through radiation treatment. The results of the study underscore the discrepancy between patient fears and actual experiences. Additionally they demonstrate a shortage of public knowledge around RT treatment and its benefits.

This discrepancy between knowledge and experience alone demonstrates room for improvement of pre‐treatment counseling. Pre‐treatment education has been shown to reduce anxiety and increase self‐efficacy in patients receiving radiation.^12^ Increased self‐efficacy has been associated with a higher quality of life.^13^ Improved education and self‐efficacy can lead to increased patient adherence to treatment, which has been directly associated with improved patient outcomes.^14^ Since it has been shown that many patients are dissatisfied with pre‐treatment education,^15^ improving this aspect of overall treatment may lead to an overall better patient experience.

Fear and concern, however, have been shown to be of the most influential factors in a patient's decision of treatment methods.^10^, ^16^ While almost half of the respondents reported hearing scary stories, almost every patient (83%) reported that the overall RT experience was less scary than predicted. Additionally, only few patients (10%) indicated being surprised by the severity of the RT side effects. More than half (57.5%) found the severity to be either slightly or significantly less than expected in the short‐term, while 60% found it to be slightly or significantly less in the long‐term. The top‐three most commonly top ranked fears of RT included organ damage (26%), skin burns (14%), and not being able to carry out normal daily activities (10%).

Regarding short‐term organ damage, most patients reported slightly or significantly less than expected anal/rectal pain (60%), urinary habit changes (52.5%), and abdominal discomfort (75%). However it should be noted that 42.5% reported slightly or significantly more than expected bowel habit changes. Regarding long‐term organ damage, many patients reported slightly or significantly less than expected anal/rectal pain (72%), bowel habit changes (40%) and problems with flatulence (45%), and “as expected” urinary habit changes (32.5%) and sexual function (47.5%).

Regarding short‐term skin burns, most patients reported slightly or significantly less than expected (68%) skin changes. Regarding long‐term skin burns, many patients reported slightly or significantly less than expected changes in appearance of area treated with radiation to be (42.5%) and dissatisfaction with body image (52.5%).

Regarding short‐term changes in not being able to carry out normal daily activities, many patients reported slightly or significantly less than expected limitations to social activities during treatment (37.5%), limitations to work activities (40%), and disruption to friends and family (37.5%). Regarding long‐term changes in normal daily activities, many patients reported slightly or significantly less than expected problems in planning activities in advance (42.5%).

In our study, patients found that the experiences of the side effects they were mostly concerned about were predominantly less than expected. This discrepancy between fear and treatment experience again highlights the opportunity for improved pre‐treatment counseling to establish realistic and consistent patient expectations. This is especially important, as it has been noted that the expectation of a side effect may have more bearing on a patient's experience than the absolute severity itself.^10^ It has been shown that psychosocial stress plays a significant role in a patient's cancer experience, and that there is an ability to ameliorate this with the support from healthcare providers.^17^ In fact, it has been demonstrated that healthcare providers are an important source of support for patients.^18^, ^19^, ^20^

Lastly, in the context of trimodality therapy, patients reported RT to be the least difficult of the treatments. Of the modalities, surgery was most commonly rated as the most difficult (50%) and most responsible for long‐term effects (55%). Chemotherapy was least commonly rated as most responsible for long‐term effects (13%). And RT was least commonly rated as the most difficult treatment (13%). This furthermore highlights the overall positive reception of RT therapy.

Despite the promising feedback from the participants, there are several limitations to this study. For instance, all of the patients despite their diversity came from the same institution. This may have biased the results due to geographic and socioeconomic influences. Additionally, the nature of this study was cross‐sectional, meaning it only allowed for a momentary assessment of these outcomes. However, there was a variety in lengths of follow‐up which provided the ability to assess perceptions with follow‐ups that ranged between ≥3 months after adjuvant chemotherapy or ≥6 months after ileostomy reversal, whichever came later. This cross‐sectional study is also a reflection of current treatment paradigms and may shift with adoptions of different treatment approaches, such as increasing chemotherapy use with increased TNT adoption and organ preservation, which our study did not assess. Recall bias also may have played a role in the responses of patients. However, there was comparative agreement across this sample of patients suggesting this bias may have had only minimal impact. Lastly, the response rate of 51% limited the data to 40 patients. It is unclear whether any of the 38 patients who did not response are no longer alive, which may have naturally limited patients who had worse outcomes and a more difficult time with treatment. Further studies are recommended for further validation of the results. It may be of benefit to have a collaborative, nationwide survey assessing multiple cancer treatment perceptions versus experiences to solidify and expand these findings. Furthermore, we recommend adding in perspective questioning during clinical trials and routine practice, as this can help mitigate potential recall bias and serve as a helpful baseline among shifts in treatment paradigms.

Nonetheless, based on all the aforementioned data, it is clear that RT in the setting of rectal cancer is a significantly more positive treatment experience than what is perceived. Keeping this in mind can be of critical importance for healthcare providers that are counseling their future patients throughout their treatment journey.

## AUTHOR CONTRIBUTIONS

**Basil H. Chaballout:** Conceptualization (equal); data curation (equal); formal analysis (equal); funding acquisition (equal); investigation (equal); methodology (equal); project administration (equal); resources (equal); software (equal); supervision (equal); validation (equal); visualization (equal); writing – original draft (lead); writing – review and editing (lead). **Eric Chang:** Conceptualization (equal); data curation (equal); formal analysis (equal); funding acquisition (equal); investigation (equal); methodology (equal); project administration (equal); resources (equal); software (equal); supervision (equal); validation (equal); visualization (equal); writing – original draft (lead); writing – review and editing (lead). **Narek Shaverdian:** Conceptualization (equal); data curation (equal); formal analysis (equal); funding acquisition (equal); investigation (equal); methodology (equal); project administration (equal); resources (equal); software (equal); supervision (equal); validation (equal); visualization (equal); writing – original draft (equal); writing – review and editing (equal). **Percy P. Lee:** Conceptualization (equal); data curation (equal); formal analysis (equal); funding acquisition (equal); investigation (equal); methodology (equal); project administration (equal); resources (equal); software (equal); supervision (equal); validation (equal); visualization (equal); writing – original draft (equal); writing – review and editing (equal). **Phillip J Beron:** Conceptualization (equal); data curation (equal); formal analysis (equal); funding acquisition (equal); investigation (equal); methodology (equal); project administration (equal); resources (equal); software (equal); supervision (equal); validation (equal); visualization (equal); writing – original draft (equal); writing – review and editing (equal). **Michael Steinberg:** Conceptualization (equal); data curation (equal); formal analysis (equal); funding acquisition (equal); investigation (equal); methodology (equal); project administration (equal); resources (equal); software (equal); supervision (equal); validation (equal); visualization (equal); writing – original draft (equal); writing – review and editing (equal). **Ann Raldow:** Conceptualization (equal); data curation (equal); formal analysis (equal); funding acquisition (equal); investigation (equal); methodology (equal); project administration (equal); resources (equal); software (equal); supervision (equal); validation (equal); visualization (equal); writing – original draft (equal); writing – review and editing (equal).

## CONFLICT OF INTEREST STATEMENT

The authors declare no conflicts of interest.

## Supporting information


Data S1.

## Data Availability

The data that support the findings of this study are available from the corresponding author upon reasonable request.
