# Supplementary material for: The patient's perspective on radiation for rectal cancer: Initial expectations versus actual experience
Source: Cancer Med. 2023 Sep 29;12(19):19978–86. doi: 10.1002/cam4.6541 (PMC10587958; doi:10.1002/cam4.6541)
Supplement: Supplementary file 1 — Data S1. [file CAM4-12-19978-s001.docx]

**Rectal Cancer Radiation Experience Questionnaire**

This questionnaire is designed to evaluate specifically **your radiation treatment experience for rectal cancer** so that we can continue to improve the quality of care that we provide our patients. All information will remain ***strictly confidential.***

Directions: Please fill in marks like this: Not like this: 🗸

**NAME:** ______________________________________________ **TODAY’S DATE:** ________________

**Question 1:**

Thinking back to when you were first diagnosed, how much did you know about radiation therapy?

O None

O Little knowledge

O Some

O A lot

**Question 2:**

If you had some or a lot of knowledge about radiation therapy, what was the main source of your information?

O Internet articles or blogs

O Scientific articles

O Friends/family

O Physicians

O Other (please specify): _________________________________

**Question 3:**

Prior to meeting your radiation doctor, had you read or heard scary stories about patients being treated with radiation and having serious side effects?

O Yes

O No

O Unsure

*Continue →*

**Question 4:**

Prior to beginning therapy, what was your biggest fear about undergoing treatment for rectal cancer?

**Question 5:**

Please **choose and rank the top three** of the following that best capture your ***initial*** ***(before receiving radiation)*** fears and worries about **radiation therapy**. (1 = Most fearful/worrisome; please only mark 3)

**1 2 3**

O O O Possible skin burning

O O O Possibly being radioactive

O O O Possible damage to my internal organs (i.e., bowel, bladder, etc.)

O O O Possible damage to my immune system

O O O Possible pain

O O O Possible nausea

O O O Possible diarrhea

O O O Possible changes to my appearance

O O O Possible changes in my ability to engage in sexual activity

O O O Possibly feeling weak

O O O Possibly feeling tired

O O O Possibly not being able to carry out my normal daily functions or work

O O O Possibly not being able to engage in my social/leisure time activities

O O O The cost of treatment

O O O Other (please specify): ______________________________________

**OR**

O N/A – I wasn’t fearful of any possibilities

*Continue →*

**Question 6: Experience with Radiation Short-Term Side-Effects:**

The following questions ask you to think back to your **expectations of the short-term side effects of radiation therapy before treatment started.** Please compare these initial expectations to what you **actually experienced during treatment.**

**For example:**

Patient Jane thought she would have very bad rectal pain during her radiation treatment. But, she actually had no pain during treatment. Therefore, Jane would select “*significantly less* than I had expected”.

**The buttock/anal/rectal pain (burning/soreness/discomfort) I experienced while undergoing radiation treatment was…**

O what I had expected

O slightly less than I had expected

O significantly less than I had expected

O slightly more than I had expected

O significantly more than I had expected

**The abdominal discomfort (pain/bloating/nausea/vomiting) I experienced while undergoing radiation treatment was…**

O what I had expected

O slightly less than I had expected

O significantly less than I had expected

O slightly more than I had expected

O significantly more than I had expected

**The skin changes (redness/darkening/itchiness) I experienced while undergoing radiation treatment were…**

O what I had expected

O slightly less than I had expected

O significantly less than I had expected

O slightly more than I had expected

O significantly more than I had expected

*Continue →*

**Question 6: Short-Term Side-Effects Continued…..**

**The changes to my bowel habits (irregularity/bleeding/leakage/urgency/increased frequency) I experienced while undergoing radiation treatment were…**

O what I had expected

O slightly less than I had expected

O significantly less than I had expected

O slightly more than I had expected

O significantly more than I had expected

**The changes to my urinary habits (pain/leakage/increased frequency) I experienced while undergoing radiation treatment were…**

O what I had expected

O slightly less than I had expected

O significantly less than I had expected

O slightly more than I had expected

O significantly more than I had expected

**The changes to my interest in sexual activity I experienced while undergoing radiation treatment were…**

O what I had expected

O slightly less than I had expected

O significantly less than I had expected

O slightly more than I had expected

O significantly more than I had expected

**The limitations in my work activities during radiation treatment were…**

O what I had expected

O slightly less than I had expected

O significantly less than I had expected

O slightly more than I had expected

O significantly more than I had expected

*Continue →*

**Question 6: Short-Term Side-Effects Continued…..**

**The limitations to my hobbies or other social/leisure time activities during radiation treatment were…**

O what I had expected

O slightly less than I had expected

O significantly less than I had expected

O slightly more than I had expected

O significantly more than I had expected

**The changes to my energy level/tiredness during radiation treatment were…**

O what I had expected

O slightly less than I had expected

O significantly less than I had expected

O slightly more than I had expected

O significantly more than I had expected

**The amount of anxiety/worry I felt during radiation treatment was…**

O what I had expected

O slightly less than I had expected

O significantly less than I had expected

O slightly more than I had expected

O significantly more than I had expected

**The amount of sadness/unhappy feelings I felt during radiation treatment was…**

O what I had expected

O slightly less than I had expected

O significantly less than I had expected

O slightly more than I had expected

O significantly more than I had expected

*Continue →*

**Question 6: Short-Term Side-Effects Continued…..**

**The disruption my radiation treatment was to the important people in my life (family/close friends) was…**

O what I had expected

O slightly less than I had expected

O significantly less than I had expected

O slightly more than I had expected

O significantly more than I had expected

**Overall, the short-term side effects I experienced during radiation treatment were…**

O what I had expected

O slightly less than I had expected

O significantly less than I had expected

O slightly more than I had expected

O significantly more than I had expected

*Continue →*

**Question 7: Experience with Radiation Long-Term Side-Effects:**

The following questions ask you to think back to your **expectations of the long-term side effects of radiation therapy before treatment started.** Please compare these initial expectations to what you **actually experience now.** Please only evaluate the long-term side effects you consider to be **the result of radiation therapy** and *not* surgery or chemotherapy.

**For example:**

Patient Jane was expecting to have permanent rectal pain. But, now a year after radiation, she actually notices only minor long-term pain. Therefore, Jane would select “*significantly less* than I had expected”.

**The amount of buttock/anal/rectal pain (burning/soreness/discomfort) I now experience as a result of radiation treatment is…**

O what I had expected

O slightly less than I had expected

O significantly less than I had expected

O slightly more than I had expected

O significantly more than I had expected

**The changes to my bowel habits (irregularity/bleeding/leakage/urgency/increased frequency) I now experience as a result of radiation treatment are…**

O what I had expected

O slightly less than I had expected

O significantly less than I had expected

O slightly more than I had expected

O significantly more than I had expected

**The changes to my urinary habits (pain/leakage/increased frequency) I now experience as a result of radiation treatment are…**

O what I had expected

O slightly less than I had expected

O significantly less than I had expected

O slightly more than I had expected

O significantly more than I had expected *Continue →*

**Question 7: Experience with Radiation Long-Term Side-Effects Continued…..**

**The problems with gas (flatulence) I now experience as a result of radiation treatment are…**

O what I had expected

O slightly less than I had expected

O significantly less than I had expected

O slightly more than I had expected

O significantly more than I had expected

**The appearance of the area treated with radiation is now…**

O what I had expected

O slightly better than I had expected

O significantly better than I had expected

O slightly worse than I had expected

O significantly worse than I had expected

**The changes to my interest in sexual activity I now experience as result of radiation therapy are…**

O what I had expected

O slightly less than I had expected

O significantly less than I had expected

O slightly more than I had expected

O significantly more than I had expected

**The changes to my sexual function (e.g., erectile dysfunction, vaginal dryness/tightness, pain during intercourse) I now experience as result of radiation therapy are…**

O what I had expected

O slightly less than I had expected

O significantly less than I had expected

O slightly more than I had expected

O significantly more than I had expected

*Continue →*

**Question 7: Experience with Radiation Long-Term Side-Effects Continued…..**

**My dissatisfaction with my body image as a result of radiation therapy is now…**

O what I had expected

O slightly better than I had expected

O significantly better than I had expected

O slightly worse than I had expected

O significantly worse than I had expected

**The problems I have planning activities in advance (e.g., meeting friends, going out of the house because I need to be close to a toilet) as result of radiation therapy are…**

O what I had expected

O slightly less than I had expected

O significantly less than I had expected

O slightly more than I had expected

O significantly more than I had expected

**The long-term changes to my energy level as result of radiation therapy are…**

O what I had expected

O slightly less than I had expected

O significantly less than I had expected

O slightly more than I had expected

O significantly more than I had expected

**Overall, the long-term side effects of radiation therapy have been…**

O what I had expected

O slightly less than I had expected

O significantly less than I had expected

O slightly more than I had expected

O significantly more than I had expected

*Continue →*

**Question 8:** Please select how strongly you would agree or disagree with the following statements.

**“Based on my actual experience, most of the negative stories about radiation therapy I read online turned out to be false.”**

O Definitely false

O Mostly false

O Neither true nor false

O Mostly true

O Definitely true

O Not applicable/I did not read stories online

**“Based on my actual experience, most of the negative stories I heard about radiation therapy from friends/family turned out to be false.”**

O Definitely false

O Mostly false

O Neither true nor false

O Mostly true

O Definitely true

O Not applicable/I did not hear stories from friends and family

**Question 9:** Please select how strongly you would agree or disagree with the following statements.

**“Overall, my radiation therapy experience was much harder than I had expected it to be.”**

O Definitely false

O Mostly false

O Neither true nor false

O Mostly true

O Definitely true

**“Overall, my radiation therapy experience was less scary than I thought it would be.”**

O Definitely false

O Mostly false

O Neither true nor false

O Mostly true

O Definitely true *Continue →*

**“Knowing what I do know, I feel I would be better off if I had chosen not to undergo radiation therapy.”**

O Definitely false

O Mostly false

O Neither true nor false

O Mostly true

O Definitely true

**Question 10:** Please select how strongly you would agree or disagree with the following statements.

**“If future patients knew the *real* truth about radiation therapy, they would be less scared about treatment.”**

O Definitely false

O Mostly false

O Neither true nor false

O Mostly true

O Definitely true

**“I was surprised by how severe my actual side-effects from radiation treatment were.”**

O Definitely false

O Mostly false

O Neither true nor false

O Mostly true

O Definitely true

**Question 11:** Please answer the following questions.

**Of your treatments for rectal cancer (surgery/radiation therapy/chemotherapy), which did you find the most difficult?**

O Surgery

O Radiation Therapy

O Chemotherapy

*Continue →*

**Of your treatments for rectal cancer (surgery/radiation therapy/chemotherapy), which did you find the least difficult?**

O Surgery

O Radiation Therapy

O Chemotherapy

**Which long-term side effects from your ENTIRE treatment (surgery, radiation therapy AND chemotherapy), for rectal cancer do you find the most bothersome?**

O Changes to my bowel habits

O Changes to my urinary habits

O Changes to my sexual function

O Changes to my energy level

O Changes to my mood

O N/A – I don’t have any bothersome long-term side effects from treatment

O Other: _______________________________________________________

**Of your treatments for rectal cancer (surgery/radiation therapy/chemotherapy), which do you feel is most responsible for your long-term side effects?**

O Surgery

O Radiation Therapy

O Chemotherapy

**Of your treatments for rectal cancer (surgery/radiation therapy/chemotherapy), which do you feel is least responsible for your long-term side effects?**

O Surgery

O Radiation Therapy

O Chemotherapy

*Continue →*

**Optional – Question 12:**

**What is the highest level of education you have completed?**

O Less than High School

O Graduated High School

O Graduated College

O Post graduate degree

**Is there anything else you would like us to know about your treatment?**

**Thank you for taking the time to complete our survey!**

Your answers will help us to improve the quality of care that we provide our patients. We greatly appreciate your time.
